# Brain protective ventilation in acute brain injury patients with use of fully automated ventilation (BRAVE): A cross-over clinical trial

**DOI:** 10.1097/EJA.0000000000002253

**Published:** 2025-08-22

**Authors:** Robin L. Goossen, Sibilla Gavinelli, Simone Dragoni, David M.P. van Meenen, Frederique Paulus, Marcus J. Schultz, Lorenzo Ball, Nicolo’ Antonino Patroniti, Chiara Robba

**Affiliations:** From the Department of Intensive Care, Amsterdam University Medical Centres, location AMC, Amsterdam, The Netherlands (RLG, DMPvM, FP, MJSS), Department of Anaesthesia and Intensive Care, IRCCS Ospedale Policlinico San Martino, Genoa, Italy (SG, SD, LB, NAP, CR), Department of Anaesthesiology, Amsterdam University Medical Centres, location AMC, Amsterdam, The Netherlands (DMPvM), Faculty of Health, ACHIEVE, centre of applied research, University of applied research, Amsterdam, the Netherlands (FP), Mahidol Oxford Tropical Medicine Research Unit (MORU), Mahidol University, Bangkok, Thailand (MJSS), Nuffield Department of Medicine, University of Oxford, Oxford, United Kingdom (MJSS), Department of Anaesthesia, General Intensive Care and Pain Management, Division of Cardiothoracic and Vascular Anaesthesia & Critical Care Medicine, Medical University of Vienna, Vienna, Austria (MJSS)

## Abstract

**BACKGROUND:**

Invasive ventilation can be challenging in acute brain injury (ABI) patients as partial pressure of carbon dioxide and oxygen need to be kept in precise optimal ranges while simultaneously applying lung–protective ventilation. Fully automated ventilation may be effective in achieving protective ventilation targets for brain and lung.

**OBJECTIVE(S):**

To compare automated ventilation to conventional ventilation for ABI patients.

**DESIGN:**

Single–centre, observational, cross–over trial.

**SETTING:**

Primary care hospital in Italy, recruiting in 2024.

**PATIENTS:**

Twenty ABI patients receiving invasive mechanical ventilation.

**METHODS:**

We performed 3 h of data collection during conventional ventilation followed by 3 h of data collection during automated ventilation.

**MAIN OUTCOME MEASURE:**

The primary endpoint was the percentage of breaths in three predefined zones of ventilatory targets, defined as optimal, acceptable and critical. The zones were based on patient–specific ranges of four measures: end–tidal carbon dioxide (EtCO_2_), peripheral oxygen saturation (SpO_2_), tidal volume (V_T_), and maximum airway pressures (*P*_max_).

**RESULTS:**

A total of 20 patients were included. With automated ventilation the proportion [range] of breaths within the optimal zone significantly increased from 2.7% [0.0 to 23.4] to 30.5% [0.9 to 66.3] (*P* < 0.001). Automated ventilation markedly decreased the proportion of breaths in the critical zone, from 16.6% [1.9 to 41.3] to 2.1% [0.5 to 7.4] (*P* < 0.001), while slightly reducing breaths in the acceptable zone from 58.1% [34.4 to 90.9] to 45.1% [25.4 to 90.8] (*P* < 0.001). Optimal breaths increased for EtCO_2_, SpO_2_, and V_T_, but declined for *P*_max_ with automation. The percentage of time spent in each ventilation zone mirrored the percentage of breaths in each zone.

**CONCLUSION:**

Automated ventilation outperformed conventional ventilation in maintaining protective ventilation targets for brain and lung in ABI patients.

**TRIAL REGISTRATION:**

Clinicaltrials.gov identifier: NCT06367816

**KEY POINTS**

- Achieving protective ventilation targets for brain and lung simultaneously in acute brain injury (ABI) patients can be challenging; fully automated ventilation may help to achieve ventilatory targets.
- This observational cross–over trial included 20 ABI patients, with 3 h of breath-by-breath data collected during conventional ventilation and 3 h during automated ventilation
- Performance was evaluated by calculating the percentage of breaths in three predefined zones (optimal, acceptable, critical) based on patient-specific ranges for end-tidal carbon dioxide (EtCO_2_), peripheral oxygen saturation (SpO_2_), tidal volume (V_T_), and maximum airway pressures (*P*_max_).
- Automated ventilation significantly increased the percentage of breaths in the optimal zone and reduced the proportion of breaths in the critical zone compared to conventional ventilation.
- Automated ventilation outperformed conventional ventilation in maintaining protective ventilation targets for brain and lung in ABI patients.

## Introduction

Invasive ventilation in patients with acute brain injury (ABI), encompassing both traumatic and non-traumatic causes such as stroke and infection, aims to prevent secondary brain damage, which is strongly associated with worse outcomes,^1^ while simultaneously adhering to lung–protective ventilation principles. In patients with ABI, the role of partial pressure of carbon dioxide (PaCO_2_) and partial pressure of oxygen (PaO_2_) in the development of secondary brain injury are well known, both exhibiting a U-shaped relationship with outcomes.^2,3^ This underscores the importance of maintaining levels within an optimal range. Although recent guidelines have offered limited recommendations for ventilation practice in ABI patients,^4^ those addressing a specific subgroup, traumatic brain injury (TBI), emphasise the regulation of PaCO_2_ to normal or moderately low levels with a very narrow target range to modulate cerebral blood flow and intracranial pressure.^5,6^ This might be particularly challenging in clinical practice, as ABI patients are particularly prone to pulmonary complications, which further complicate their care.^7^

The use of automated ventilation modes in the Intensive Care Unit (ICU) has gained increasing attention due to their ability to streamline patient management, potentially reducing workload, and improving outcomes.^8^ Among these, the fully automated mode INTELLiVENT-Adaptive Support Ventilation (ASV) (Hamilton Medical, Bonaduz, Switzerland) is able to adjust key ventilator settings, such as tidal volume (V_T_), respiratory rate (RR), fraction of inspired oxygen (FiO_2_), and positive end-expiratory pressure (PEEP), on a breath-by-breath basis to maintain target levels of end-tidal carbon dioxide (EtCO_2_) and peripheral oxygen saturation (SpO_2_). Crucially, INTELLiVENT-ASV includes an algorithm tailored for brain-injured patients, ensuring tighter control of SpO_2_ and restricting permissive hypercapnia together with automated PEEP adjustments, to avoid potentially harmful effects.

Closed-loop ventilation modes, like INTELLiVENT-ASV, have been shown to be effective in achieving lung-protective ventilation in various groups of patients,^8^ including those at risk for or suffering from acute respiratory distress syndrome (ARDS)^9–11^ and chronic obstructive pulmonary disease (COPD).^12,13^ Despite the inclusion of some ABI patients in these studies, the effectiveness of INTELLiVENT-ASV in providing protective ventilation for both lung and brain in this patient category have not been thoroughly investigated.^14,15^

Given that ABI patients require careful management of both lung function and intracranial physiology,^16,17^ it is crucial to explore whether automated ventilation can effectively balance these dual needs. We aimed to evaluate the effectiveness of the automated mode INTELLiVENT-ASV compared to conventional ventilation in achieving protective ventilation targets for brain and lung in invasively ventilated ABI patients. We hypothesised that automated ventilation would outperform conventional ventilation in maintaining protective ventilation ranges of EtCO_2_, SpO_2_, V_T_, and maximum airway pressures (*P*_max_), for brain and lung in ABI patients.

## Methods

### Ethics

The study protocol received ethical approval from the Ethical Committee of Genova University Hospitals, Genova, Italy (N.NET-Liguria: 552/2023-DB Id 13509,) on 16 January 2024 and was registered on ClinicalTrials.gov (identifier: NCT06367816). Requirement for individual patient informed consent was obtained according to local rules. The study was conducted according to the STrengthening the Reporting of OBservational studies in Epidemiology (STROBE) checklist.

### Study design and patients

The BRAVE study was an observational, investigator-initiated, single-centre, cross-over trial conducted at the ICU of a teaching hospital in Genoa, Italy. The manufacturer of the ventilator was not involved in the study. Patients were recruited from the 1st April to the 1st October 2024.

We recruited patients >18 years of age receiving invasive ventilation on the ICU with a diagnosis of ABI (<24 h between brain injury and intubation) who were ventilated with a mechanical ventilator providing both conventional and automated ventilation with an expected duration of ventilation >24 h.

We excluded patients from this analysis if they had any contraindication to INTELLiVENT-ASV as specified by the manufacturer, or were moribund or facing end of life. Inclusion occurred as soon as possible following clinical stabilisation.

### Standard of care

The decision on the mode of ventilation, including both conventional and automated ventilation, was made by the responsible clinician. Physicians were responsible for adjusting ventilator settings and arterial blood gases (ABG) were frequently performed. All attending physicians were qualified to use this ventilator and the modes applied. ICU care, including ventilatory management, followed local policies and currently available protocols and guidelines.^5,18^ This consisted of lung–protective ventilation strategies (V_T_ of 6 to 8 ml kg^−1^ of predicted body weight (PBW), maintaining plateau pressure <27 cmH_2_O and driving pressure <15 cmH_2_O), sedation with propofol (3 to 6 mg kg^−1^ h^−1^) and/or midazolam (0.03 to 0.2 mg kg^−1^ h^−1^) and fentanyl (0.1 to 0.8 μg kg^−1^ min^−1^), and a head elevated position at 30°. PEEP was applied using a low-PEEP strategy starting at 4 to 5 cmH_2_O in ABI patients without lung injury, with adjustments targeting normoxia and tight control of EtCO_2_ and PaCO_2_ while aiming for low to moderate tidal volume of 6 to 8 ml kgPBW^−1^. Slight increases of V_T_ were tolerated if the EtCO_2_ and PaCO_2_ were poorly controlled. In cases of concurrent lung and brain injury, PEEP was increased stepwise under neuromonitoring (intracranial pressure ‘ICP’, near–infrared spectroscopy, and/or transcranial doppler as needed), aiming for a stricter control of V_T_ but still prioritising PaCO_2_ control. In obese patients requiring higher PEEP levels due to co-existing lung injury, PEEP was evaluated on a case-by-case basis, and pronation under strict neuromonitoring was preferred over PEEP levels increasing 10 to 12 cmH_2_O. Intracranial hypertension was managed using a stepwise approach based on the latest Seattle algorithm,^6^ including specific targets of O_2_ and CO_2_.

### Study protocol

In patients included in this study, and where the physician had decided to apply automated ventilation, as per protocol conventional ventilation was recorded initially for 3 h, followed by one hour of ‘wash-out’ time (no data recording), before switching subsequently to 3 h of data recording during automated ventilation. Conventional ventilation was recorded first for two reasons. First, conventional ventilation is the mode most applied at Ospedale San Martino, minimising the workload for physicians in participating in this study. Second, we know from previous studies that when automated ventilation is applied first, following the switch to conventional ventilation, clinical staff tend to set the ventilator by copying the resulting ventilation settings of the automated mode, leaving a reflection of settings of the automated mode.^19^

Based on clinical measurements, such as ICP and ABG analysis, the clinician formulated a personalised target of EtCO_2_ and SpO_2_ at the start of data collection of both conventional and automated ventilation.

At start of the conventional block, the clinician was asked whether he/she desired to make alterations to the ventilator settings with the target of EtCO_2_ and SpO_2_ in mind. During the 3 h of data collection, care, including alterations in ventilator settings, was given as usual.

At the start of the automated block, the physician set INTELLiVENT-ASV according to protocol based on the formulated targets. In all, the patient's condition ‘brain injury’ was enabled, and as a result, PEEP was set manually by the responsible clinician. When setting the target EtCO_2_, the amount of dead space ventilation, reflected by the ‘observed gap’ between the exhaled CO_2_ measured by the ventilator and the arterial partial pressure of CO_2_ (PaCO_2_) derived from the ABG, was considered, according to user instructions. After the initiation of INTELLiVENT-ASV, minute ventilation, pressure levels, and FiO_2_, were automatically adjusted by the ventilator to provide invasive ventilation based on target ranges of EtCO_2_ and SpO_2_. During the 3 h of data collection, care, including alterations in ventilatory setting, followed our clinical practice. Staffing consisted of a dedicated nurse and physician for each patient, with a technician available onsite.

### Data collection

Patient details and baseline characteristics were collected at the time of inclusion. Data included age, sex, height, weight, and medical history. Baseline characteristics encompassed the type of brain injury, neurological assessment at first evaluation, typically performed prehospital by paramedics, the presence of neurological deterioration before intubation, pupil size, and glasgow coma scale (GCS) score. Neurological deterioration was defined as a spontaneous decrease in GCS, motor score of >1 compared to previous examination with new loss of pupillary reactivity, development of pupillary asymmetry of ≥2 mm, and deterioration in neurological or CT status sufficient to warrant immediate medical or surgical intervention. Additional baseline data included the location of intubation and the reason for initiating ventilatory support. Granular breath-by-breath ventilator and gas exchange data were collected for 3 h in each mode of ventilation (automated *vs.* nonautomated ventilation), using a storage device connected to the ventilator as in previous studies performed by our team.^1,2^

At the start and end of each 3 h study block data from ABG analysis, heart rate and mean arterial pressure (MAP) were collected. If ABG analysis was not possible for clinical reasons, the most recent analysis was used.

During follow-up the following data were collected to give an accurate description of the study cohort: date of weaning from mechanical ventilation, date of last day in the ICU and in hospital, and if succumbed, date of death with a maximum of 90 days. Data collection was performed by the first, second, and/or third authors, who were not part of the clinical team.

### Study endpoints

The primary outcome was the percentage of breaths classified into three predefined ventilation zones: ‘optimal’, ‘acceptable’ and ‘critical’. These zones were based on a modified version of previously established criteria^20–22^ and defined using four measures: patients-specific target EtCO_2_ (mmHg) at the start of both conventional and automated ventilation, patient-specific target SpO_2_ (%) at the start of both conventional and automated ventilation, V_T_ (ml kgPBW^−1^) and maximum airway pressure (*P*_max_, cmH_2_O) (Table 1).

**Table 1:** Zones of ventilation used to define the primary outcome

	Optimal zone	Acceptable zone	Critical zone
EtCO_2_ (mmHg)	±2.5 from target	±>2.5 & ≤5.5 from target	<–5.5 or >5.5 from target or
SpO_2_ (%)	±1.5 from target	±>1.5 & ≤3 from target	<–3 or >3 from target or
V_T_ (ml kgPBW^−1^)	≤8	>8 & ≤12	>12 or
*P*_max_ (cmH_2_O)	≤30	>30 & ≤36	>36
Definition	All must be present	If not in the optimal zone and none of the critical zone is present	If any present

EtCO_2_, end-tidal carbon dioxide; SpO_2_, saturation of peripheral oxygen; V_T_, tidal volume; PBW, predicted body weight; *P*_max_, maximum airway pressure.

If any of the four variables was missing, zone was missing.

Secondary endpoints included the percentage of breaths within each ventilatory zone for each of the four measures, including a subdivision for EtCO_2_ and SpO_2_ of the ‘acceptable’ and ‘critical’ zone of ventilation into high and low (‘higher range acceptable’, ‘lower range acceptable’, ‘higher range critical’ and ‘lower range critical’), and the percentage of time within the three zones of ventilation.

### Definitions and calculations

We calculated dynamic driving pressure (Δ*P*) and dynamic respiratory system compliance (*C*_RS_) for each breath where the ventilator did not detect a spontaneous breath, using *P*_max_ and PEEP, as inspiratory and expiratory holds were not performed. Additionally, we computed mechanical power of ventilation (MP), fractional dead space, and ventilatory ratio (VR).

The following equations were applied:

Δ*P* (cmH_2_O) = *P*_max_ − PEEP

C_RS_ (ml cmH_2_O^−1^) = V_T_/Δ*P*

MP (J min^−1^) = 0.098 × V_T_ × RR × (*P*_max_ − 0.5 × Δ*P*)

Fractional dead space = (PaCO_2_ − EtCO_2_)/PaCO_2_

VR = (minute ventilation × PaCO_2_)/(PBW × 100 × 37.5), where minute ventilation is calculated as V_T_ × RR.

### Sample size calculation

Based on a previous study with a comparable primary endpoint,^1^ a statistical power of 0.9, *α* of 0.05, considering 10% dropout, and aiming to detect 15% difference, yielding 72 000 analysable breaths, we set a sample size of 20 patients.

### Statistical analysis

Continuous data were expressed as mean ± standard deviation or median [IQR] as appropriate. Categorical data were presented as numbers with percentages. Breaths with missing data for one or more of the four quality variables were excluded from analysis. Similarly, breaths during blocks assigned to the alternate mode were also excluded.

Ventilation characteristics for each mode were summarised by calculating patient-level means of all breaths during each ventilation block. Group-level comparisons were presented as the median [IQR] of these patient-level means. For outcomes assessing proportions of breaths, the percentage of breaths in each ventilation zone (optimal, acceptable and critical) was calculated for each patient as a percentage of their total breaths.

To compare the distribution of breaths between zones (optimal *vs.* not optimal, acceptable *vs.* not acceptable, critical *vs.* not critical) between ventilation modes, a mixed-effect logistic regression model was used. The model included all breaths categorised into binary outcomes as the dependent variable, patient as a random effect, and ventilation mode as a fixed effect. Results were reported as estimates with corresponding 95% CI and *P*-values.

For the outcome assessing percentage of time, the duration of each breath was calculated based on the RR (breaths per minute). Hence the percentage of time spent in each ventilation zone (optimal, acceptable, and critical) was calculated for each patient as a percentage of the total time of the observation period. To analyse differences in the percentage of time spent in each zone, a Beta regression model with a logit link function was used.

All analyses were performed using R-studio, version 4.3.2.

## Results

### Patient characteristics

**Fig. 1:**
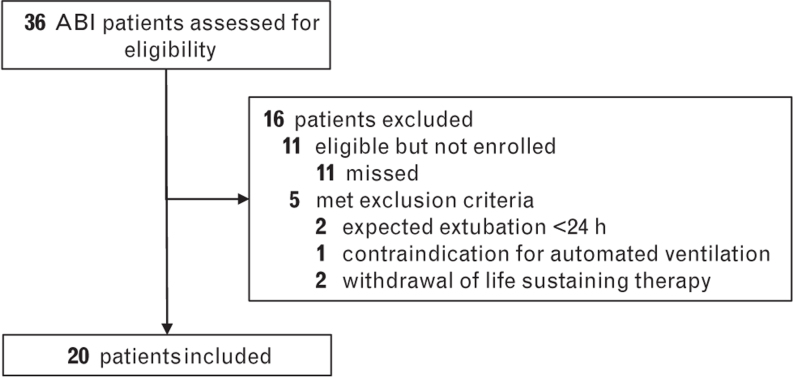
Patient flow.

Of 36 patients with ABI assessed for eligibility, we included a total of 20. The main reasons for exclusion were expected extubation <24 h and withdrawal of life sustaining therapy (Fig. 1). An even number of males and females were included with the most common type of ABI being intracranial haemorrhage (35%) followed by subarachnoidal haemorrhage (30%). The median GCS at first evaluation was 7 [3 to 12]. Most patients were intubated by the prehospital care team, primarily due to decreased consciousness or neurological deterioration. Hospital mortality was 45% (Table 2).

ABI, acute brain injury.

**Table 2:** Patient data and clinical outcomes

	(*n* = 20)
Baseline	
Age (years)	67 [61 to 72]
Sex (male)	10 (50)
Height (cm)	169 [162 to 175]
Weight (kg)	76 [70 to 86]
BMI (kg m^−2^)	28 [24 to 30]
Medical history	
Diabetes	3 (15)
Hypertension	14 (70)
Heart failure	20 (100)
Chronic kidney disease	1 (5)
COPD	20 (100)
Active haematological malignancy	1 (5)
Active solid tumour malignancy	1 (5)
Immunosuppression	20 (100)
Brain injury	
Type	
Intracranial haemorrhage	7 (35)
Subarachnoid haemorrhage	6 (30)
Traumatic brain injury	3 (15)
Ischaemic cerebrovascular incident	1 (5)
Meningo-encephalitis	1 (5)
Other	2 (10)
Neurological assessment at first evaluation	
Pupils at first evaluation	
Reactivity to light	
Both	12 (75)
One	0 (0)
None	4 (25)
Pupil size at first evaluation	
Isocoric	13 (65)
Nonisocoric	7 (35)
Neurodeterioration before intubation	
No deterioration	4 (20)
Spontaneous decrease in GCS motor score of >1 compared to previous examination	2 (10)
New loss of pupillary reactivity	0 (0)
Development of pupillary asymmetry ≥2 mm	0 (0
Deterioration in neurological or CT status sufficient to warrant immediate medical or surgical intervention	14 (70)
GCS	7 [3 to 12]
Eyes	2 [1 to 4]
Verbal	1 [1 to 3]
Motor	5 [1 to 5]
Ventilatory characteristics	
Location of intubation	
In hospital	7 (35)
Other hospital	4 (20)
Outside of the hospital	9 (45)
Reason for ventilatory support	
Respiratory failure	0 (0.0)
Cardiac arrest	0 (0.0)
Postoperative ventilation	2 (10)
Decreased consciousness	17 (85)
Coma	4 (23.5)
Neurodeterioration	12 (70.6)
Cough failure	0 (0)
Loss of airway protection	1 (5.9)
Muscular weakness	0 (0)
Airway protection	0 (0)
Other	1 (5)
Time from ICU admission to inclusion (days)	2 [1 to 4]
Clinical outcomes	
ICU mortality	8 (40)
Hospital mortality	9 (45)
28-day mortality	8 (40)
90-day mortality	9 (45)
ICU length of stay (days) in survivors	17 [9 to 28]
Hospital length of stay (days) in survivors	41 [25 to 53]
Duration of ventilation (days) in survivors	7 [6 to 9]

Numbers are presented as n (%) or median [IQR].

BMI, body mass index; COPD, chronic obstructive pulmonary disease; CT, computed tomography; GCS, Glasgow coma scale; ICU, intensive care unit.

### Ventilation characteristics

The median time from ICU admission to data collection was two days. In the conventional mode of ventilation, pressure support ventilation was the most commonly used mode. During automated ventilation a higher FiO_2_ was used, more spontaneous breaths occurred and Δ*P* was lower (Table 3).

**Table 3:** Ventilation characteristics

	Conventional ventilation	Automated ventilation	*P* value
Mode (% of breaths)			
Duopap	14.2		
PCV	40		
PSV	45.8		
INTELLiVENT-ASV		100	
V_T_ (ml kgPBW^−1^)	7.7 [6.8 to 8.6]	7.9 [6.6 to 8.4]	0.77
RR (breaths min^−1^)	15 [14 to 20]	15 [14 to 17]	0.65
Spontaneous RR (breaths min^−1^)	1 [0 to 3]	14 [9 to 15]	0.002
*P*_max_ (cmH_2_O)	22 [18 to 25]	19 [17 to 20]	0.07
PEEP (cmH_2_O)	8 [6 to 8]	8 [6 to 8]	0.96
FiO_2_ (%)	31 [30 to 36]	43 [40 to 50]	0.05
SpO_2_ (%)	99 [98 to 99]	99 [97 to 100]	0.37
ΔP (cmH_2_O)	13.9 [12.8 to 17.8]	12.0 [11.3 to 12.1]	0.04
C_RS_ (ml cmH_2_O^−1^)	32.7 [31.5 to 39.9]	35.4 [25.4 to 38.7]	0.87
MP(J min^−1^)	10.8 [7.3 to 13.9]	8.6 [5.7 to 10.0]	0.19
Calculated data at start and end of each block^a^			
Ventilatory ratio, start	1.1 [1.0 to 1.3]	1.3 [1.1 to 1.4]	
Ventilatory ratio, end	1.3 [1.1 to 1.5]	1.3 [1.1 to 1.5]	
Dead space fraction, start	0.05 [0.00 to 0.11]	0.05 [0 to 0.11]	
Dead space fraction, end	0.03 [0.00 to 0.08]	0.05 [0 to 0.14]	
*P*aO_2_/FiO_2_ (mmHg), start	290 [191 to 358]	282 [189 to 413]	
*P*aO_2_/FiO_2_ (mmHg), end	274 [226 to 329]	275 [186 to 366]	

Numbers are presented as *n* and median [IQR]. For V_T_, RR, spontaneous RR, *P*_max_, PEEP, FiO_2_, SpO_2_, driving pressure, C_RS_ and MP the mean was calculated for each block and compared between patients.

a Since calculations involved blood gas analyses, these were only calculated for the start and end of each block.

Δ*P*, driving pressure; *C*_RS_, respiratory system compliance; Duopap, dual positive airway pressure; FiO_2_, fraction of inspired oxygen; MP, mechanical power; PaO_2_, partial pressure of oxygen; PBW, predicted body weight; PCV, pressure controlled ventilation; PEEP, Positive end-expiratory pressure; PSV, pressure support ventilation; RR, respiratory rate; *P*_max_, maximum airway pressure; SpO_2_, saturation of peripheral oxygen; V_T_, tidal volume.

### Primary endpoint

**Fig. 2:**
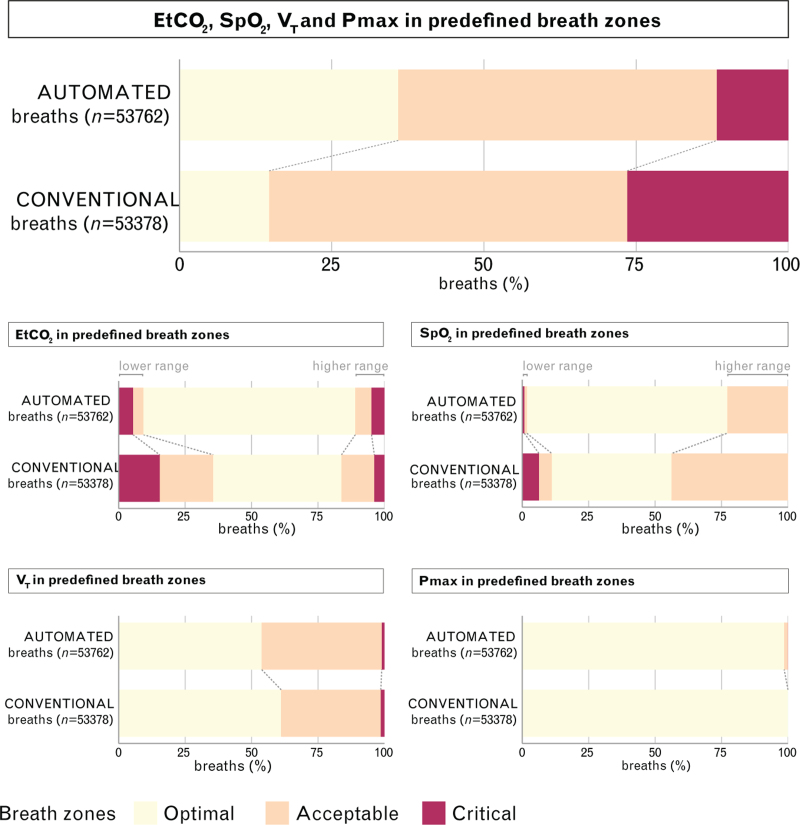
Mean percentage of breaths in predefined zones of ventilation.

During conventional ventilation, the median percentage of breaths classified within the optimal zone was 2.7% [0.0 to 23.4], which increased to 30.5% [0.9 to 66.3] during automated ventilation (*P* < 0.001). Automated ventilation also resulted in a lower median percentage of breaths in the acceptable zone (from 58.1% [34.4 to 90.9] to 45.1% [25.4 to 90.8], *P* < 0.001) and the critical zone (from 16.6% [1.9 to 41.3] to 2.1% [0.5 to 7.4], *P* < 0.001) compared to conventional ventilation (Table 4 and Fig. 2).

**Table 4:** Outcomes

	Conventional ventilation	Automated ventilation	Estimate	*P* value
Primary outcome				
% of breaths in the optimal zone	2.7 [0 to 23.4]	30.5 [0.9 to 66.3]	1.79 (1.75 to1.83)	<0.001
% of breaths in the acceptable zone	58.1 [34.4 to 90.9]	45.1 [25.4 to 90.8]	−0.53 (−0.56 to −0.50)	<0.001
% of breaths in the critical zone	16.6 [1.9 to 41.3]	2.1 [0.5 to 7.4]	−1.64 (−1.69 to −1.60)	<0.001
Secondary outcomes				
EtCO_2_				
% of breaths in optimal zone	57.9 [9.3 to 77.8]	90.1 [77.7 to 98.8]	2.26 (2.22 to 2.30)	<0.001
% of breaths in acceptable zone	30.4 [7.6 to 39.6]	8.3 [1.3 to 14.9]	−1.83 (−1.87 to −1.80)	<0.001
% of breaths in critical zone	2.3 [0 to 24.2]	0.6 [0 to 5.6]	−1.21 (−1.26 to −1.16)	<0.001
SpO_2_				
% of breaths in optimal zone	44.5 [4.0 to 79.8]	93.4 [57.4 to 97.2]	2.43 (2.39 to 2.47)	<0.001
% of breaths in acceptable zone	41.6 [16.2 to 96.0]	5.9 [2.5 to 37.5]	−2.22 (−2.26 to −2.18)	<0.001
% of breaths in critical zone	0 [0 to 0.7]	0 [0 to 0.7]	−2.30 (−2.30 to −2.30)	<0.001
V_T_				
% of breaths in optimal zone	62.3 [32.0 to 93.3]	55.8 [11.2 to 92.6]	−0.49 (−0.53 to −0.46)	<0.001
% of breaths in acceptable zone	33.2 [4.6 to 67.6]	42.9 [7.3 to 88.5]	0.52 (0.49 to 0.55)	<0.001
% of breaths in critical zone	0.6 [0.1 to 2.1]	0.5 [0 to 1.2]	−0.29 (−0.41 to −0.17)	<0.001
*P*_max_				
% of breaths in optimal zone	100 [100 to 100]	99.9 [98.4 to 100]	−5.1 (−6.11 to −4.31)	<0.001
% of breaths in acceptable zone	0 [0 to 0]	0.1 [0.0 to 1.6]	5.1 (4.30 to 6.09)	<0.001
% of breaths in critical zone	0 [0 to 0]	0.0 [0.0 to 0.0]	52.9 (29.2 to 76.6)	<0.001
Time				
% of time in the optimal zone	2.7 [0 to 23.2]	30.5 [0.9 to 66.3]	0.65 (−0.11 to 1.41)	0.09
% of time in the acceptable zone	58.5 [34.1 to 91.0]	40.3 [23.2 to 90.8]	0.06 (−0.66 to 0.79)	0.86
% of time in the critical zone	17.2 [2.1 to 37.9]	2.3 [0.5 to 7.5]	−0.90 (−1.60 to −0.19)	0.01

Numbers are presented as median [IQR] or estimate (95% CI).

EtCO_2_, end-tidal carbon dioxide; *P*_max_, maximum airway pressure; SpO_2_ saturation of peripheral oxygen; V_T_, tidal volume.

Breaths (*n*) represents the total number of breaths from all included patients for conventional and automated ventilation, respectively. Lower and higher ranges of acceptable and critical breaths were only assessed for EtCO_2_ and SpO_2_. EtCO_2_, end-tidal carbon dioxide; SpO_2_, saturation of peripheral oxygen; V_T_, tidal volume; *P*_max_, maximum airway pressure.

### Secondary endpoints

#### Subcategories of ventilation zones: EtCO_2_, SpO_2_, V_T_, *P*_max_

During automated ventilation the percentage of optimal breaths for target EtCO_2_ increased compared to conventional ventilation. The percentage of both acceptable and critical breaths were reduced, with the most significant reduction observed in the lower range. However, in the higher range, the percentage of critical breaths showed a slight increase (Fig. 2 and Table 4).

When comparing automated ventilation to conventional ventilation in terms of target SpO_2_, the percentage of optimal breaths increased, while the percentages of acceptable and critical breaths decreased (Fig. 2 and Table 4).

Regarding V_T,_ the percentage of optimal breaths decreased, and acceptable breaths increased during automated ventilation (Fig. 2 and Table 4).

With respect to *P*_max_, all breaths were classified as optimal during conventional ventilation. In contrast, during automated ventilation, a minority of breaths were classified as acceptable and critical (Fig. 2 and Table 4).

#### Time

**Fig. 3:**
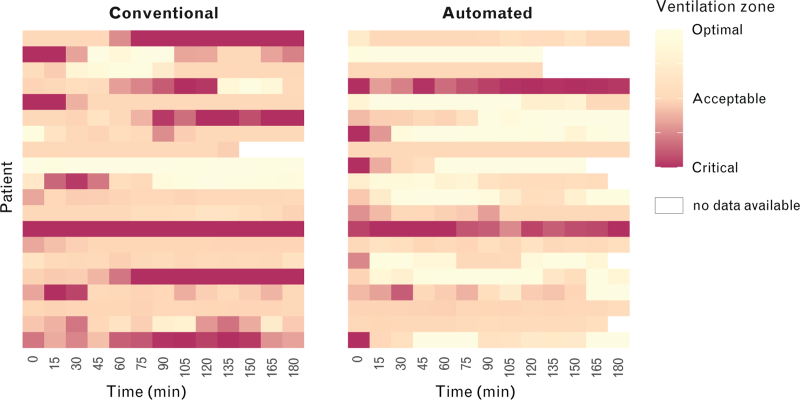
Heat map displaying ventilation zones on a continuous scale for every 15 min after the start of data collection.

Patients during automated ventilation spent a higher median duration of time in the optimal zone and a lower median duration of time in the acceptable and critical zones compared to conventional ventilation, as illustrated in the heat map, showing the quality of breaths in consecutive blocks of 15 min (Table 4 and Fig. 3).

Each row represents one patient. Each breath was categorised as optimal (1), acceptable (2), or critical (3), with the mean value for each 15-min period representing the colour of the corresponding block. Breath data was not available for some blocks due to clinical reasons (white).

## Discussion

In this study, we found that automated ventilation increased the percentage of breaths within optimal ranges for ABI patients, while reducing the risk of injurious tidal volumes compared to conventional ventilation.

To our knowledge, this is the first study which used highly granular breath-by-breath data during automated ventilation, including all ABI patients, and taking in consideration personalised EtCO_2_ and SpO_2_ targets, and protective ventilation settings. The predefined primary outcome captures both the efficacy of ventilation tailored to brain-injured patients and its safety in terms of lung protection, which is particularly important given the elevated risk of pulmonary complications in ABI patients.^23^ This approach is consistent with previous studies on automated ventilation,^20,21^ yet we are the first to incorporate patient-specific targets, highly relevant for ABI patients when categorising breaths. Moreover, our study generated high-resolution data, providing valuable insights while maintaining a pragmatic protocol.

Our findings align with those of a previous study that compared INTELLiVENT-ASV to conventional ventilation using hourly measurements in a subset of 12 ABI patients with TBI. This research reported reduced variability in EtCO_2_ and PaCO_2_ with automated ventilation. However, it did not feature patient-specific targets of EtCO_2_ and SpO_2_.^15^ Additionally, our results partially align with findings from two studies that used the same primary endpoint and high-granular data in cardiac surgery patients.^20,21^ The study most comparable to ours, regarding the criteria to define breath zones, found a higher percentage of optimal breaths and a lower percentage of acceptable and critical breaths with INTELLiVENT-ASV.^21^ While this study applied the same definitions for breath zones, it categorised EtCO_2_ based on actual values rather than patient-specific targets. Notably, in patients with ABI, staying within the target range of EtCO_2_ is even more crucial due to its potential impact on the cerebrovascular system. Therefore, while the optimal zone had a similar range width in both studies, our study applied a narrower acceptable range, leading to more breaths being classified as critical. The same applies to SpO_2_, which helps explain some of the observed differences in the actual percentages of optimal, acceptable and critical breaths. An unexpected difference was observed in V_T_ since we used the same ranges to define V_T_ zones. The cardiac surgery study reported a greater proportion of breaths where V_T_ was categorised as optimal during automated ventilation compared to conventional ventilation whereas our study observed a decrease in the proportion of optimal breaths with automated ventilation. This difference may reflect variations in the ventilation strategies required for these distinct patient groups or could be attributed to practical factors, such as the accurate input of patient height during the initiation of automated ventilation.

Even though the percentage of breaths in the optimal EtCO_2_ zone increased significantly with automated ventilation, the proportion of critical breaths in the higher range also increased, an outcome that may be undesirable in patients with ABI. Current management algorithms for TBI patients recommend maintaining normal or moderately low PaCO_2_ levels to avoid exacerbating ICP.^6^ However, we also know that PaCO_2_ exhibits a U-shaped relationship with mortality in ABI patients, and forced hypocapnia has even been shown to be associated with a higher hazard ratio than hypercapnia.^2^ Future research should explore the relationship between EtCO_2_, PaCO_2_, and their subsequent impact on ICP and mortality in ABI patients ventilated with automated ventilation, to be able to judge if this proves an undesirable effect of automated ventilation in this group.

Even though the percentage of breaths within optimal range of V_T_ was lower during automated ventilation, the median V_T_ PBW^−1^ did not differ between the two modes, and Δ*P* was lower with automated ventilation. Traditionally, relatively high tidal volumes have been used in patients with ABI to prevent hypercapnia, cerebral vasodilation, and intracranial hypertension.^24^ While recent evidence supports low tidal volume ventilation (LTVV) in patients without ARDS^25,26^ a nuanced approach may be necessary in ABI patients. One meta–analysis^27^ found no survival benefit of LTVV in ABI patients, while a more recent RCT reported worse outcomes with a protective ventilatory strategy.^28^ The authors suggested this may be due to higher PaCO_2_ values causing cerebral vasodilation or elevated PEEP impairing cerebral venous drainage. Despite this, literature indicates that LTVV can still play a role in preventing pulmonary complications in ABI patients.^29,30^ For example, factors known to contribute to ventilator-induced lung injury (VILI) in ARDS, such as *P*_max_, Δ*P* and MP, have also been associated with VILI in ABI patients.^31–33^ Our findings indicated a shift from a higher proportion of breaths in the optimal V_T_ zone to the acceptable zone. In this light, this shift might not be problematic for ABI patients.

Altogether, this study supports the use of fully automated ventilation modes in patients with ABI. However, future research should explore whether these modes offer an intracerebral protective effect, extending benefits beyond the quality of breaths. Specifically, it is important to assess how closely the arterial targets (PaCO_2_ and PaO_2_) are achieved, as these are indirectly targeted through EtCO_2_ and SpO_2_ during automated ventilation. In some patients in our study, invasive ICP monitoring was in place, and clinicians appropriately targeted lower PaCO_2_ levels in response to elevated ICP, following current guidelines.^6^ During automated ventilation, EtCO_2_ targets were adjusted to reflect this; however, this applied to a minority of patients, as invasive ICP monitoring was not routinely used. This highlights the need for future studies combining automated ventilation with comprehensive neuromonitoring, to better understand how targeting arterial CO_2_ and O_2_ levels via end–tidal or peripheral measurements may affect intracerebral conditions such as cerebral oxygenation and ICP.^14^ Additionally, the role of PEEP in ABI patients remains a subject of debate.^34^ In this study, the ‘brain injury’ patient category was enabled, resulting in manual PEEP titration and disabling PEEP automation. Given evolving evidence suggesting that permissive hypercapnia may not be strictly contraindicated in ABI patients,^2^ bypassing this patient–specific setting might even further improve the quality of breaths, or even neurophysiological status. Finally, future studies should also consider the implications for clinical workload and cost-effectiveness, as these are critical factors in the broader adoption of automated ventilation systems.

This study has several limitations. Firstly, while data was deliberately first collected during conventional ventilation followed by automated ventilation to prevent known carryover effects from automated to conventional ventilation,^35^ we could not adjust for potential carryover effects in the opposite direction. Additionally, patient conditions or respiratory status, and, consequently, the difficulty of achieving the set target PaCO_2_ and PaO_2,_ may have changed over time, potentially affecting the results. Secondly, missing data predominantly occurred at the end of the automated ventilation block due to clinical logistics, such as transport for CT scans. While end-of-block PaCO_2_ and *P*aO_2_ measurements were included, the sample size was insufficient to determine whether automated ventilation more effectively achieved arterial targets compared to conventional ventilation. Thirdly, the conventional ventilation group included a range of commonly used modes (PCV, PSV and Duopap), which reflects real-world ICU practice. This heterogeneity limits direct comparisons with individual conventional modes, and the study was not powered to detect differences between specific ventilatory strategies. Nonetheless, the finding that fully automated ventilation outperformed clinician-selected modes, despite their variability, supports the generalisability and clinical relevance of our results. Lastly, the use of surrogate endpoints does not necessarily predict improved clinical outcomes. Future randomised clinical trials of this fully automated ventilation mode in ABI patients should investigate patient-centred outcomes.

## Conclusion

Automated ventilation outperformed conventional ventilation in maintaining brain and lung–protective ventilation ranges for ABI patients.^18^
